# Relationships of habitual daily alcohol consumption with all-day and time-specific average glucose levels among non-diabetic population samples

**DOI:** 10.1265/ehpm.22-00215

**Published:** 2023-03-16

**Authors:** Maho Ishihara, Hironori Imano, Isao Muraki, Kazumasa Yamagishi, Koutatsu Maruyama, Mina Hayama-Terada, Mari Tanaka, Mikako Yasuoka, Tomomi Kihara, Masahiko Kiyama, Takeo Okada, Midori Takada, Yuji Shimizu, Tomotaka Sobue, Hiroyasu Iso

**Affiliations:** 1 Department of Social Medicine, Osaka University Graduate School of Medicine, Suita, Osaka, Japan; 2 Osaka Center for Cancer and Cardiovascular Disease Prevention, Osaka Joto-ku, Osaka, Japan; 3 Department of Public Health Medicine, Faculty of Medicine, and Health Services Research and Development Center, University of Tsukuba, Tsukuba, Ibaraki, Japan; 4 Ibaraki Western Medical Center, Chikusei, Ibaraki, Japan; 5 Department of Bioscience, Graduate School of Agriculture, Ehime University, Matsuyama, Ehime, Japan; 6 Department of Public Health, Kindai University Faculty of Medicine, Osakasayama, Osaka, Japan; 7 Yao City Public Health Center, Yao, Osaka, Japan; 8 Department of Frailty Research, Research Institute, National Center for Geriatrics and Gerontology, Obu, Aichi, Japan; 9 Institute for Global Health Policy Research, Bureau of International Health Cooperation, National Center for Global Health and Medicine, Shinjuku, Tokyo, Japan

**Keywords:** Glucose levels, Alcohol consumption, Time-specific, Community-based samples, Non-diabetic, Flash glucose monitoring system

## Abstract

**Background:**

Alcohol consumption is a prevalent behavior that is bi-directionally related to the risk of type 2 diabetes. However, the effect of daily alcohol consumption on glucose levels in real-world situations in the general population has not been well elucidated. This study aimed to clarify the relationship between alcohol consumption and all-day and time-specific glucose levels among non-diabetic individuals.

**Methods:**

We investigated 913 non-diabetic males and females, aged 40–69 years, during 2018–2020 from four communities across Japan. The daily alcohol consumption was assessed using a self-report questionnaire. All-day and time-specific average glucose levels were estimated from the interstitial glucose concentrations measured using the Flash glucose monitoring system for a median duration of 13 days. Furthermore, we investigated the association between all-day and time-specific average glucose levels and habitual daily alcohol consumption levels, using never drinkers as the reference, and performed multiple linear regression analyses after adjusting for age, community, and other diabetes risk factors for males and females separately.

**Results:**

All-day average glucose levels did not vary according to alcohol consumption categories in both males and females. However, for males, the average glucose levels between 5:00 and 11:00 h and between 11:00 and 17:00 h were higher in moderate and heavy drinkers than in never drinkers, with the difference values of 4.6 and 4.7 mg/dL for moderate drinkers, and 5.7 and 6.8 mg/dL for heavy drinkers. Conversely, the average glucose levels between 17:00 and 24:00 h were lower in male moderate and heavy drinkers and female current drinkers than in never drinkers; the difference values of mean glucose levels were −5.8 for moderate drinkers, and −6.1 mg/dL for heavy drinkers in males and −2.7 mg/dL for female current drinkers.

**Conclusions:**

Alcohol consumption was associated with glucose levels in a time-dependent biphasic pattern.

## Background

The prevalence of diabetes mellitus (DM) and impaired glucose tolerance is rapidly increasing worldwide. According to the International Diabetes Federation (IDF), the worldwide diabetic population reached 537 million in 2021, indicating that one in ten adults has diabetes mellitus [1]. Longstanding high blood glucose levels can damage blood vessels, leading to various health problems, such as retinopathy, renal disease, peripheral neuropathy [2], coronary heart disease, stroke [3, 4], and deaths attributable to diabetes mellitus. Healthcare expenditure due to diabetes mellitus is also on the rise, creating a significant social, financial, and healthcare system burden worldwide [5, 6]. Therefore, establishing evidence-based preventive measures against diabetes mellitus is an urgent issue.

Alcohol consumption is a prevalent behavior that is bi-directionally related to the risk of type 2 diabetes. Light drinking could lower this risk, whereas heavy drinking could increase it [7–9]. However, the effect of alcohol consumption on glucose metabolism in daily life has not been well elucidated. A clinical experimental study of five non-diabetic young adult males showed that the ingestion of 48 g ethanol reduced glycogenesis by 45%, although plasma glucose concentrations did not change [10]. A randomized controlled trial of 51 non-diabetic postmenopausal females demonstrated that the ingestion of 30 g/day ethanol for eight weeks lowered fasting serum insulin concentrations by 20% but did not affect fasting plasma glucose levels compared with those in the placebo group [11]. However, these previous experimental studies did not capture the impact of daily alcohol consumption on glucose levels in real-world situations in the general population. Therefore, we aimed to clarify the relationship between habitual daily alcohol consumption and all-day and time-specific glucose levels in daily life using a Flash glucose monitoring system in community-based non-diabetic samples.

## Methods

### Study subjects

We included 1260 non-diabetic individuals (377 males and 883 females) who provided consent to participate in this study, were aged 40–69 years, and hailed from four communities, namely Ikawa town, Akita Prefecture (a northwestern rural community); Minami-Takayasu district, Yao City, Osaka Prefecture (a midwestern suburb); Kyowa district, Chikusei City, Ibaraki Prefecture (a mideastern rural community); and Kamisu City, Ibaraki Prefecture (an industrial area), in Japan. The first three communities were sub-cohorts of the Circulatory Risk in Communities Study (CIRCS), an ongoing dynamic cohort study on lifestyle-related diseases involving approximately 12000 [12]. In Kamisu City, approximately 8000 adults receive health checkups annually, and the subjects were selected from among them. Surveys of daily glucose monitoring were conducted in 2019 in Ikawa, 2018–2019 in Minami-Takayasu, 2018–2020 in Kyowa, and 2019–2020 in Kamisu.

**Fig. 1:**
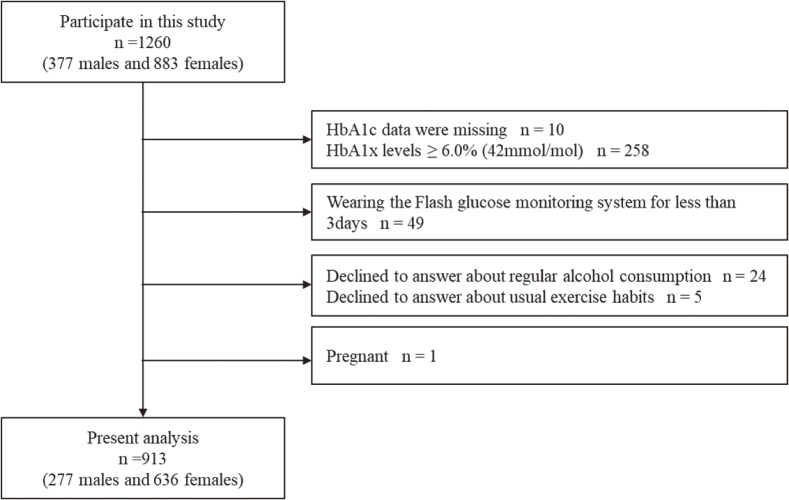
Flowchart of study participants of the present study

We excluded those participants whose hemoglobin A1c (HbA1c) data were missing (n = 10), whose HbA1c levels were ≥6.0% (42 mmol/mol) (n = 258), who were wearing the Flash glucose monitoring system for less than 3 days (n = 49), who declined to answer about regular alcohol consumption (n = 24), who declined to answer about usual exercise habits (n = 5), or who were pregnant (n = 1). In total, 913 participants (277 males and 636 females) were included in the present analysis (Fig. 1).

### Measurements

Each participant’s habitual daily alcohol consumption was assessed using a questionnaire. Participants were asked combined questions 1. whether they drank alcohol, and for current drinkers, 2. the amount of alcohol consumed per day in *go*-units (a Japanese traditional unit of volume equivalent to 23 g of ethanol). Alcohol consumption was categorized into five groups (never, former, Light; current <23, Moderate; 23–45, and Heavy; ≥46 g/day ethanol) for males and into three groups (never, former, and Current) for females.

The following covariates were also elicited by the questionnaire: the number of cigarettes smoked per day, their medical history, family history of diabetes mellitus, frequency and duration per occasion of physical activity during the past year (namely, for frequency: <1 /month, 1–3 /month, 1–2 /week, 3–4 /week, and every day; for time, <30 minutes, 30–59 minutes, 1–2 hours, 2–3 hours, and ≥4 hours), and skipping breakfast (yes or no).

Blood was collected from the participants in plastic serum separator gel tubes. The serum was allowed to stand for 15–20 minutes after collection and centrifuged for 15 minutes at 3000 rpm in a centrifuge with a turning radius of 16 to 18 cm within 30 minutes. In Ikawa and Minami-Takayasu, serum samples were transported to the Osaka Center for Cancer and Cardiovascular Disease Prevention, and serum glucose levels were measured using the hexokinase and glucose-6-phosphate dehydrogenase methods and an automatic analyzer (TBA-2000FR; Toshiba, Otawara, Tochigi, Japan), whereas HbA1c levels were measured using high-performance liquid chromatography with an HLC-723 G8 (Tosoh, Minato-ku, Tokyo, Japan). In kyowa and Kamisu, serum samples were transported to the Ibaraki Health Service Association, and serum glucose levels were measured using the hexokinase and ultraviolet absorption spectrophotometric methods, while HbA1c levels were assessed via enzymatic methods using an automatic analyzer (JCA-BM9130; JEOL Ltd, Tokyo, Japan). The body mass index (BMI) was calculated as weight in light clothing (kg) divided by height squared in stocking feet (m^2^). The systolic and diastolic blood pressure levels were measured in the right arm by trained observers according to the unified epidemiological method, using automatic sphygmomanometers, except for participants in Minami-Takayasu in 2019, for whom standard mercury sphygmomanometers were used.

### Measurement of the daily glucose levels

**Fig. 2:**
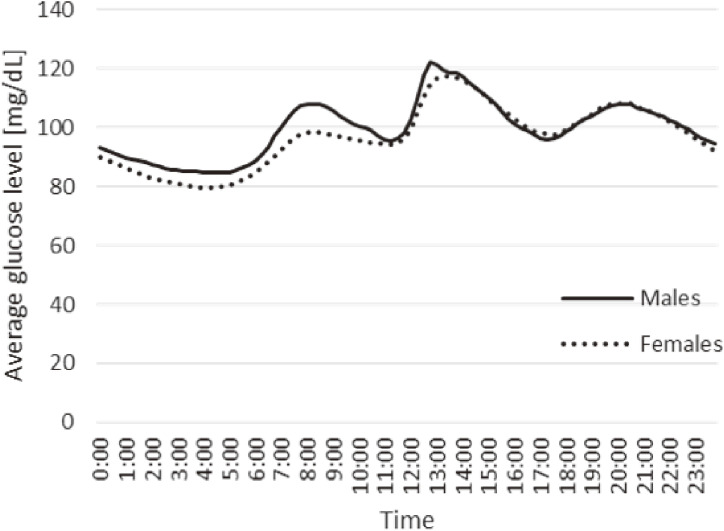
Time-specific glucose levels among males and females.

Glucose concentrations in the interstitial fluid were measured every 15 min for up to 15 days using a Flash glucose monitoring system (FGM; FreeStyle Libre Pro System, Abbott Diabetes Care, Inc. Alameda, CA, USA) in the upper left arm. We monitored glucose continuously, and analyzed date from day 2 through to the second-to-last day of recording. This was done because Freestyle Libre Pro is reported to be less accurate on the first day of measurement [13]. In addition, fewer data could be collected on the first and last days; hence, the average glucose values could not be calculated reliably. Finally, because of differences in the number of days of measurement among participants, the analyzed data amounted to one day in the shortest case and 13 days in the longest case. The distribution of the participants who had worn the FGM sensor for each span of days is shown in Table 1. For time-of-day classifications, because the nadirs of the daily glucose levels were found at 0:00, 5:00, 11:00, and 17:00 h (Fig. 2), we categorized the time range as follows: all-day, 0:00 to 5:00, 5:00 to 11:00, 11:00 to 17:00, and 17:00 to 24:00 h. The time-specific average glucose levels in each alcohol consumption category were defined as the averaged values of individual mean glucose levels, which were calculated by averaging the measurement data of each participant in each time range without adjustment for sex, age, and community.

**Table 1:** The distribution of the number of days participants wore the FGM sensor.

	**Alcohol consumption**				

	**Never**	**Former**	**Light** **<23**	**Moderate** **23–45**	**Heavy** **≥46**
Males					
No. of participants, n	77	13	77	51	59
Days wearing FGM sensor					
1 day, %	2.6	0.0	1.3	2.0	1.7
2 to 3 days, %	9.1	15.4	3.9	7.8	6.8
4 to 6 days, %	5.2	0.0	2.6	3.9	5.1
7 to 12 days, %	13.0	38.5	14.3	15.7	23.7
13 days (full days), %	70.1	46.2	77.9	70.6	62.7


	**Never**	**Former**	**Current**		

Females					
No. of participants, n	399	42	195		
Days wearing FGM sensor					
1 day, %	2.0	2.4	1.0		
2 to 3 days, %	1.8	4.8	3.6		
4 to 6 days, %	3.8	4.8	5.1		
7 to 12 days, %	18.1	19.1	14.4		
13 days (full days), %	74.4	69.1	75.9		

### Statistical analysis

We compared the time-specific average glucose levels according to usual alcohol consumption with reference to never drinking, using multiple linear regression for males and females separately. The adjustment values included age (continuous), community (Ikawa, Minami-Takayasu, Chikusei, Kamisu), and other confounding variables, such as habitual smoking (never, former, current), the BMI (continuous), habitual physical activity (exercise at least 30 minutes a day at least once a week, yes or no), family history of diabetes mellitus (yes or no), and skipping breakfast (yes or no). We conducted further adjustment for HbA1c levels at screening, after which HbA1c was categorized into two groups (≤5.5 and 5.6–5.9%, ≤37 and 38–41 mmol/mol) and stratified the HbA1c groups.

All analyses were performed using SAS version 9.4 (SAS Institute, Cary, NC, USA). P-values < 0.05 were considered to indicate statistical significance in two-tailed analyses.

## Results

Of all participants, 667 (73%) wore the FGM sensor for full days. Table 2 shows the baseline characteristics according to the habitual alcohol consumption category among males and females. In males who were moderate and heavy alcohol consumers, the systolic and diastolic blood pressure, fasting and nonfasting blood glucose levels at screening, and the average glucose levels between 5:00 and 11:00 h and between 11:00 and 17:00 h were higher than those in others. Male heavy drinkers smoked more than others. Similarly, females who were current drinkers had higher fasting and nonfasting blood glucose levels as well as increased proportions of current smoking and skipping breakfast.

**Table 2:** Characteristics of study subjects at baseline according to alcohol consumption.

	**Alcohol consumption**									

	**Never**		**Former**		**Light** **<23**		**Moderate** **23–45**		**Heavy** **≥46**	
Males										
No. of participants, n	77		13		77		51		59	
Age, years	55.7	(9.1)	58.4	(8.9)	56.5	(9.1)	58.7	(8.0)	56.3	(9.1)
Average glucose level, mg/dL										
All-day	100.5	(10.6)	98.6	(13.4)	98.2	(10.7)	101.5	(9.4)	99.3	(12.2)
0:00 to 5:00 h	88.9	(11.6)	85.1	(12.3)	85.6	(12.1)	89.8	(11.6)	86.7	(12.6)
5:00 to 11:00 h	97.6	(11.9)	94.7	(14.0)	97.1	(11.7)	102.7	(11.3)	100.3	(15.2)
11:00 to 17:00 h	106.9	(12.1)	106.2	(16.7)	105.3	(12.5)	112.0	(10.5)	114.4	(14.7)
17:00 to 24:00 h	105.9	(12.1)	105.0	(14.4)	101.9	(13.1)	99.9	(10.3)	97.2	(12.2)
HbA1c at screening, %	5.5	(0.2)	5.6	(0.2)	5.6	(0.2)	5.6	(0.2)	5.5	(0.3)
HbA1c at screening, mmol/mol	37	(2.6)	38	(2.4)	38	(2.5)	38	(2.2)	37	(2.6)
≤5.5 (≤37 mmol/mol), %	48.1		38.5		45.5		52.5		60.0	
5.6–5.9 (38–41 mmol/mol), %	52.0		61.5		54.6		47.5		40.0	
Fasting glucose at screening*, mg/dL	94.2	(6.9)	93.2	(8.1)	93.7	(6.8)	96.4	(9.5)	97.3	(9.1)
Non-fasting glucose at screening*, mg/dL	95.4	(12.6)	94.0	(11.6)	99.0	(16.1)	97.9	(16.6)	107.1	(28.7)
Body mass index, kg/m^2^	23.7	(3.3)	23.9	(2.4)	23.8	(3.2)	24.1	(2.6)	23.5	(3.1)
Waist Circumstance, cm	84.2	(9.6)	84.1	(7.6)	84.4	(9.3)	85.6	(7.4)	84.5	(8.6)
Systolic blood pressure, mmHg	122.8	(14.0)	122.2	(13.4)	124.6	(13.5)	130.6	(15.0)	130.9	(14.9)
Diastolic blood pressure, mmHg	77.1	(9.9)	79.5	(9.9)	78.0	(9.6)	83.4	(9.0)	83.1	(9.6)
Antihypertensive medication, %	18.2		23.1		24.7		29.4		27.1	
Smoking habit, %										
never	32.5		15.4		29.9		13.7		6.8	
past	44.2		76.9		52.0		66.7		61.0	
current	23.4		7.7		18.2		19.6		32.2	
Exercise, %	39.0		61.5		41.6		47.1		37.3	
Family history of diabetes mellitus, %	6.5		15.4		6.5		2.0		13.6	
Skipping breakfast, %	26.0		7.7		18.2		15.7		22.0	


	**Never**		**Former**		**Current**					

Females										
No. of participants, n	399		42		195					
Age, years	56.5	(8.4)	53.1	(8.3)	54.7	(8.0)				
Average glucose level, mg/dL										
All-day	97.6	(10.8)	93.5	(12.7)	95.9	(10.6)				
0:00 to 5:00 h	83.4	(11.5)	80.7	(14.2)	82.9	(11.4)				
5:00 to 11:00 h	92.8	(11.6)	88.6	(13.1)	92.7	(11.8)				
11:00 to 17:00 h	107.4	(13.0)	102.6	(13.4)	106.6	(13.7)				
17:00 to 24:00 h	103.5	(12.4)	98.9	(14.2)	98.7	(12.1)				
HbA1c at screening, %	5.6	(0.2)	5.6	(0.2)	5.5	(0.3)				
HbA1c at screening, mmol/mol	37	(2.5)	38	(2.1)	37	(2.9)				
≤5.5 (≤37 mmol/mol), %	41.6		30.2		48.2					
5.6–5.9 (38–41 mmol/mol), %	58.4		69.8		51.8					
Fasting glucose at screening*, mg/dL	91.4	(6.8)	92.0	(6.6)	93.6	(9.1)				
Non-fasting glucose at screening*, mg/dL	92.5	(13.2)	90.8	(10.1)	96.4	(17.1)				
Body mass index, kg/m^2^	22.3	(3.6)	22.8	(4.5)	22.4	(3.7)				
Waist Circumstance, cm	79.0	(10.2)	80.5	(11.3)	79.7	(11.1)				
Systolic blood pressure, mmHg	120.9	(17.3)	116.6	(16.4)	119.8	(15.3)				
Diastolic blood pressure, mmHg	73.1	(10.5)	71.6	(9.9)	74.1	(10.4)				
Antihypertensive medication, %	10.3		7.0		5.1					
Smoking habit, %										
never	83.7		60.5		62.6					
past	10.3		30.2		22.6					
current	6.0		9.3		14.9					
Exercise, %	45.4		34.9		40.0					
Family history of diabetes mellitus, %	9.0		32.6		12.8					
Skipping breakfast, %	13.5		7.0		19.0					

In parentheses: standard deviations.

**Fig. 3:**
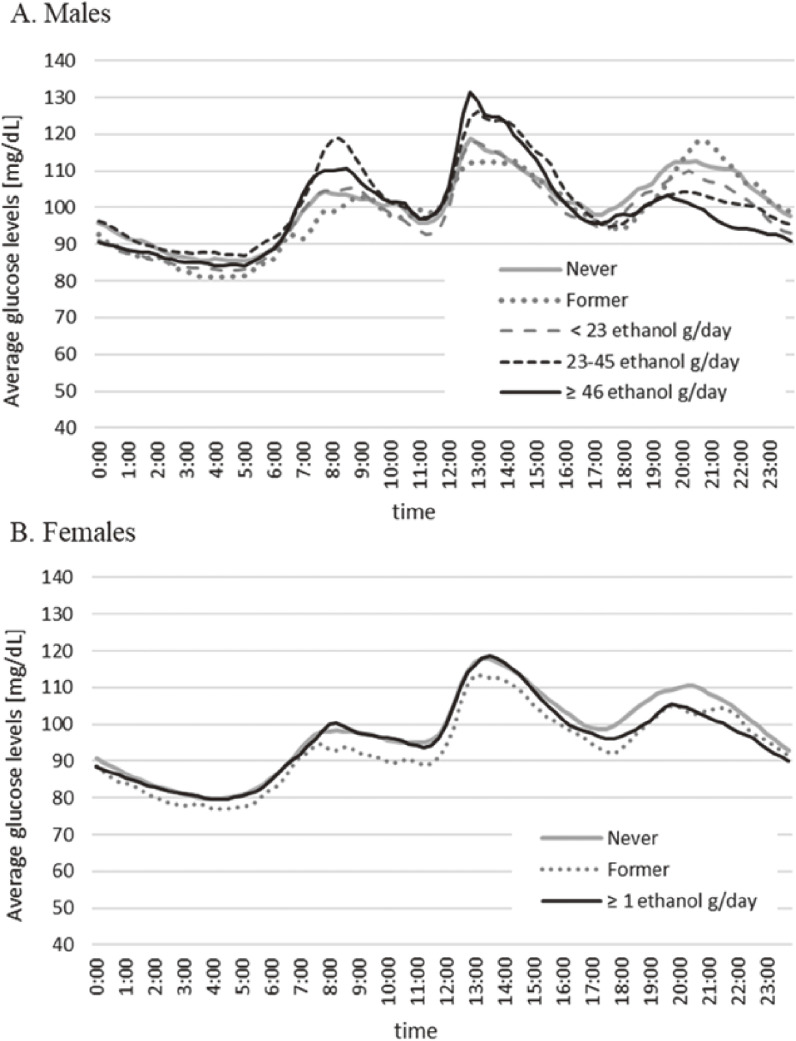
Time-specific glucose levels according to alcohol consumption category among males and females.

Figure 3 shows the time-specific variations of average glucose levels among alcohol consumption categories, and Table 3 shows the time-specific predicted differences in average glucose levels according to alcohol consumption categories among males and females. The all-day average glucose levels did not vary among alcohol consumption categories in either males or females. Among males, the time-specific average glucose levels between 5:00 and 11:00 h and between 11:00 and 17:00 h were approximately 5 to 7 mg/dL higher in moderate and heavy drinkers than in never drinkers, according to model 3 of the multivariable models. Contrastingly, the time-specific average glucose levels between 17:00 and 24:00 h were 6 mg/dL lower in male moderate and heavy drinkers and 3 mg/dL lower in female drinkers than in never drinkers, according to model 3.

**Table 3:** Associations of time-specific average glucose levels (mg/dL) with alcohol consumption category.

	**Alcohol consumption**											

	**Never**	**Former**		**Light** **<23**			**Moderate** **23–45**			**Heavy** **≥46**		
		**β (95%CI)**	**P-value**	**β (95%CI)**	**P-value**		**β (95%CI)**	**P-value**		**β (95%CI)**	**P-value**	
Males												
No. of participants, n	77	13		77			51			59		
all-day												
model 1	Ref.	3.4(−2.8,9.5)	.281	−1.8(−5.0,1.4)	.258		1.8(−1.8,5.4)	.336		0.8(−2.7.4.3)	.647	
model 2	Ref.	2.9(−3.2,9.1)	.348	−1.9(−5.1,1.3)	.236		1.0(−2.7,4.7)	.596		0.4(−3.2,3.9)	.832	
model 3	Ref.	3.0(−3.0,9.0)	.322	−2.0(−5.1,1.1)	.200		0.9(−2.7,4.5)	.613		1.4(−2.1,4.8)	.444	

0:00 to 5:00 h												
model 1	Ref.	1.4(−5.5,8.3)	.697	−2.6(−6.2,1.0)	.152		2.5(−1.6,6.6)	.236		−0.3(−4.2,3.6)	.879	
model 2	Ref.	1.6(−5.2,8.3)	.644	−2.5(−6.0,1.0)	.155		1.4(−2.6,5.5)	.486		−0.7(−4.6,3.1)	.706	
model 3	Ref.	1.7(−4.9,8.2)	.621	−2.6(−6.0,0.8)	.131		1.4(−2.6,5.3)	.498		0.2(−3.6,4.0)	.910	

5:00 to 11:00 h												
model 1	Ref.	2.4(−4.7,9.5)	.508	0.0(−3.7,3.7)	.997		5.7(1.5,10.0)	.008	†	4.8(0.7,8.8)	.021	*
model 2	Ref.	1.8(−5.4,9.0)	.620	−0.3(−4.0,3.5)	.888		4.7(0.4,9.0)	.031	*	4.3(0.2,8.5)	.039	*
model 3	Ref.	1.9(−5.0,8.8)	.587	−0.4(−4.0,3.2)	.826		4.6(0.5,8.8)	.028	*	5.7(1.6,9.7)	.006	†

11:00 to 17:00 h												
model 1	Ref.	4.5(−2.8,11.8)	.225	−1.2(−4.9,2.6)	.550		5.5(1.2,9.8)	.013	*	6.5(2.4,10.7)	.002	†
model 2	Ref.	3.4(−3.9,10.8)	.360	−1.4(−5.2,2.4)	.465		4.8(0.4,9.2)	.034	*	6.0(1.8,10.2)	.006	†
model 3	Ref.	3.5(−3.8,10.8)	.346	−1.5(−5.3,2.3)	.436		4.7(0.4,9.1)	.034	*	6.8(2.5,11.0)	.002	†

17:00 to 24:00 h												
model 1	Ref.	4.6(−2.3,11.5)	.193	−3.4(−7.0,0.1)	.060		−5.4(−9.4,−1.3)	.010	*	−6.7(−10.6,−2.7)	.001	†
model 2	Ref.	4.5(−2.5,11.4)	.210	−3.4(−7.0,0.2)	.066		−5.8(−10.0,−1.6)	.007	†	−7.0(−11.0,−3.0)	.001	†
model 3	Ref.	4.5(−2.4,11.4)	.196	−3.5(−7.0,0.1)	.056		−5.8(−10.0,−1.7)	.006	†	−6.1(−10.1,−2.1)	.003	†


	**Never**	**Former**		**Current**								
		**β (95%CI)**	**P-value**	**β (95%CI)**	**P-value**							

Females												
No. of participants, n	399	42		195								
all-day												
model 1	Ref.	1.2(−2.0,4.4)	.454	−0.3(−1.9,1.4)	.762							
model 2	Ref.	1.2(−2.1,4.4)	.479	−0.2(−1.9,1.5)	.791							
model 3	Ref.	0.6(−2.6,3.7)	.710	−0.1(−1.7,1.6)	.937							

0:00 to 5:00 h												
model 1	Ref.	1.0(−2.7,4.7)	.588	0.3(−1.7,2.2)	.790							
model 2	Ref.	0.8(−2.8,4.4)	.655	−0.2(−2.0,1.7)	.874							
model 3	Ref.	0.3(−3.2,3.9)	.852	0.0(−1.9,1.9)	.987							

5:00 to 11:00 h												
model 1	Ref.	1.0(−2.5,4.4)	.583	1.3(−0.5,3.1)	.162							
model 2	Ref.	0.7(−2.9,4.2)	.705	1.4(−0.4,3.3)	.130							
model 3	Ref.	0.2(−3.3,3.7)	.920	1.6(−0.2,3.4)	.090							

11:00 to 17:00 h												
model 1	Ref.	1.6(−2.2,5.5)	.406	1.0(−1.1,3.0)	.356							
model 2	Ref.	1.7(−2.3,5.6)	.412	1.2(−0.9,3.3)	.259							
model 3	Ref.	1.1(−2.8,5.0)	.590	1.4(−0.7,3.4)	.189							

17:00 to 24:00 h												
model 1	Ref.	1.2(−2.4,4.8)	.518	−3.0(−4.9,−1.1)	.002	†						
model 2	Ref.	1.4(−2.3,5.1)	.450	−2.9(−4.9,−1.0)	.003	†						
model 3	Ref.	0.7(−2.8,4.3)	.683	−2.7(−4.6,−0.9)	.005	†						

β, partial regression coefficient; CI, confidence Intervals;

Model 1 was adjusted for age and community. Model 2 was adjusted further for smoking habit, BMI, family history of diabetes mellitus, exercise habits, skipping breakfast. Model 3 was adjusted further for HbA1c.

* P < 0.05, † P < 0.01, ‡ P < 0.001

Table 4 shows the results stratified by HbA1c levels at screening. The higher time-specific average glucose levels between 5:00 and 11:00 h and between 11:00 and 17:00 h in male heavy drinkers were more evident among males with 5.6–5.9% (38–41 mmol/mol) HbA1c levels. The lower time-specific average glucose levels between 17:00 and 24:00 h in male moderate and heavy drinkers and female drinkers were more evident among individuals with HbA1c ≤ 5.5% (≤37 mmol/mol). The lower time-specific average glucose levels between 17:00 and 24:00 h in male light drinkers were more evident among males with HbA1c levels of 5.6–5.9% (38–41 mmol/mol).

**Table 4:** Associations of time-specific average glucose levels with alcohol consumption category, stratified by HbA1c levels.

	**Alcohol consumption**											

	**Never**	**Former**		**Light** **<23**			**Moderate** **23–45**			**Heavy** **≥46**		
		**β (95%CI)**	**P-value**	**β (95%CI)**	**P-value**		**β (95%CI)**	**P-value**		**β (95%CI)**	**P-value**	
Males												
No. of participants, n	37	5		35			20			31		
HbA1c ≤ 5.5%												
all-day												
model 1	Ref.	1.8(−8.2,11.8)	.718	0.1(−4.4,4.6)	.952		−0.2(−5.8,5.4)	.943		−0.9(−5.7,3.9)	.724	
model 2	Ref.	−0.2(−10.4,10.0)	.965	−0.4(−4.9,4.1)	.863		−2.5(−8.2,3.3)	.396		−3.0(−8.1,2.1)	.246	
model 3	Ref.	−0.8(−10.8,9.2)	.880	−1.0(−5.4,3.5)	.665		−2.9(−8.5,2.7)	.313		−2.1(−7.2,2.9)	.398	

0:00 to 5:00 h												
model 1	Ref.	−3.6(−14.7,7.6)	.531	−2.2(−7.3,2.8)	.384		−4.7(−11.0,1.5)	.138		−2.9(−8.2,2.5)	.296	
model 2	Ref.	−5.7(−16.8,5.4)	.313	−2.4(−7.4,2.5)	.328		−7.3(−13.5,−1.1)	.022	*	−4.9(−10.4,0.6)	.083	
model 3	Ref.	−6.4(−17.1,4.4)	.244	−3.2(−8.0,1.6)	.191		−7.8(−13.9,−1.8)	.012	*	−3.8(−9.2,1.6)	.166	

5:00 to 11:00 h												
model 1	Ref.	2.6(−9.0,14.2)	.660	1.7(−3.5,7.0)	.511		4.6(−1.9,11.0)	.168		3.9(−1.8,9.5)	.176	
model 2	Ref.	−0.2(−12.2,11.8)	.974	0.7(−4.6,6.0)	.799		2.1(−4.7,8.8)	.544		1.6(−4.3,7.6)	.585	
model 3	Ref.	−0.8(−12.6,11.0)	.895	0.0(−5.2,5.3)	.988		1.6(−5.0,8.2)	.629		2.6(−3.3,8.5)	.388	

11:00 to 17:00 h												
model 1	Ref.	3.4(−9.0,15.8)	.591	1.6(−4.0,7.2)	.564		5.6(−1.3,12.6)	.112		4.6(−1.4,10.6)	.128	
model 2	Ref.	1.1(−11.5,13.8)	.859	0.9(−4.7,6.5)	.758		3.5(−3.6,10.5)	.336		2.1(−4.1,8.4)	.503	
model 3	Ref.	0.8(−11.8,13.4)	.904	0.5(−5.2,6.1)	.868		3.2(−3.9,10.3)	.376		2.7(−3.6,9.0)	.397	

17:00 to 24:00 h												
model 1	Ref.	3.6(−7.4,14.5)	.519	−0.9(−5.8,4.1)	.727		−6.1(−12.2,0.0)	.051		−8.1(−13.4,−2.9)	.003	†
model 2	Ref.	2.4(−8.8,13.5)	.676	−1.0(−5.9,4.0)	.702		−8.0(−14.3,−1.7)	.013	*	−10.0(−15.5,−4.4)	.001	‡
model 3	Ref.	1.9(−9.2,12.9)	.740	−1.5(−6.4,3.4)	.541		−8.4(−14.6,−2.2)	.008	†	−9.1(−14.7,−3.6)	.001	†

Hb A1c 5.6%–5.9%												
No. of participants, n	40	8		42			31			28		
all-day												
model 1	Ref.	3.6(−4.3,11.6)	.368	−3.8(−8.3,0.7)	.094		2.3(−2.7,7.2)	.364		2.9(−2.2,7.9)	.267	
model 2	Ref.	3.6(−4.6,11.9)	.387	−3.8(−8.4,0.8)	.108		2.2(−2.9,7.2)	.397		2.9(−2.4,8.1)	.281	
model 3	Ref.	3.3(−4.9,11.6)	.426	−3.7(−8.3,0.9)	.113		2.0(−3.0,7.1)	.425		2.9(−2.3,8.1)	.270	

0:00 to 5:00 h												
model 1	Ref.	4.2(−4.8,13.1)	.358	−3.0(−8.1,2.0)	.236		6.7(1.2,12.2)	.018	*	2.3(−3.3,8.0)	.418	
model 2	Ref.	4.4(−4.5,13.3)	.331	−3.0(−7.9,2.0)	.234		6.3(0.9,11.8)	.023	*	1.8(−3.8,7.5)	.520	
model 3	Ref.	4.2(−4.7,13.1)	.354	−2.9(−7.9,2.0)	.242		6.3(0.8,11.7)	.025	*	1.9(−3.8,7.5)	.512	

5:00 to 11:00 h												
model 1	Ref.	2.2(−7.1,11.5)	.643	1.8(−7.1,3.4)	.488		5.9(0.2,11.6)	.044	*	6.8(0.9,12.7)	.025	*
model 2	Ref.	2.2(−7.4,11.8)	.652	−1.6(−6.9,3.8)	.564		5.8(−0.1,11.7)	.054		7.2(1.1,13.2)	.021	*
model 3	Ref.	1.7(−7.8,11.1)	.725	−1.4(−6.7,3.8)	.590		5.6(−0.2,11.3)	.060		7.3(1.3,13.2)	.018	*

11:00 to 17:00 h												
model 1	Ref.	4.3(−5.1,13.7)	.368	−3.9(−9.2,1.4)	.144		4.5(−1.2,10.3)	.122		8.8(2.8,14.7)	.004	†
model 2	Ref.	3.8(−5.9,13.6)	.438	−4.0(−9.4,1.4)	.147		4.2(−1.7,10.2)	.162		9.1(3.0,15.3)	.004	†
model 3	Ref.	3.6(−6.1,13.3)	.467	−3.9(−9.3,1.5)	.153		4.1(−1.8,10.1)	.172		9.2(3.0,15.3)	.004	†

17:00 to 24:00 h												
model 1	Ref.	3.9(−5.4,13.2)	.403	−6.1(−11.3,−0.8)	.023	*	−6.0(−11.7,−0.2)	.041	*	−5.2(−11.1,0.7)	.083	
model 2	Ref.	4.1(−5.5,13.8)	.402	−6.1(−11.4,−0.7)	.027	*	−5.7(−11.6,0.2)	.058		−5.4(−11.5,0.7)	.080	
model 3	Ref.	3.8(−5.8,13.5)	.432	−6.0(−11.3,−0.6)	.028	*	−5.8(−11.7,0.1)	.053		−5.4(−11.5,0.7)	.082	


	**Never**	**Former**		**Current**								
		**β (95%CI)**	**P-value**	**β (95%CI)**	**P-value**							

Females												
No. of participants, n	166	13		94								
HbA1c ≤ 5.5%												
all-day												
model 1	Ref.	2.4(−2.6,7.3)	.352	0.3(−1.8,2.5)	.771							
model 2	Ref.	2.5(−2.6,7.6)	.333	0.2(−2.0,2.5)	.841							
model 3	Ref.	2.2(−2.9,7.3)	.391	0.4(−1.8,2.6)	.708							

0:00 to 5:00 h												
model 1	Ref.	0.5(−5.4,6.4)	.871	1.8(−0.7,4.4)	.156							
model 2	Ref.	0.3(−5.6,6.2)	.922	1.4(−1.2,4.0)	.278							
model 3	Ref.	0.1(−5.8,6.0)	.974	1.6(−1.0,4.2)	.239							

5:00 to 11:00 h												
model 1	Ref.	1.7(−3.9,7.3)	.559	1.3(−1.2,3.7)	.310							
model 2	Ref.	1.7(−4.1,7.4)	.565	1.4(−1.1,3.9)	.259							
model 3	Ref.	1.5(−4.3,7.2)	.616	1.6(−0.9,4.1)	.216							

11:00 to 17:00 h												
model 1	Ref.	4.0(−2.2,10.3)	.208	2.0(−0.8,4.7)	.158							
model 2	Ref.	4.4(−2.0,10.8)	.176	2.1(−0.7,4.9)	.142							
model 3	Ref.	4.1(−2.3,10.4)	.206	2.3(−0.5,5.0)	.108							

17:00 to 24:00 h												
model 1	Ref.	2.9(−2.8,8.6)	.320	−3.0(−5.5,−0.5)	.019	*						
model 2	Ref.	3.2(−2.6,9.0)	.282	−3.3(−5.8,−0.7)	.013	*						
model 3	Ref.	2.8(−3.0,8.5)	.348	−3.0(−5.5,−0.4)	.021	*						

HbA1c 5.6%–5.9%												
No. of participants, n	233	29		101								
all-day												
model 1	Ref.	0.2(−3.8,4.2)	.917	−0.2(−2.6,2.2)	.896							
model 2	Ref.	0.0(−4.2,4.2)	.999	−0.1(−2.5,2.3)	.931							
model 3	Ref.	−0.2(−4.4,4.0)	.925	−0.2(−2.6,2.2)	.867							

0:00 to 5:00 h												
model 1	Ref.	0.7(−4.0,5.4)	.782	−0.5(−3.3,2.3)	.735							
model 2	Ref.	−0.4(−5.0,4.2)	.868	−0.9(−3.6,1.8)	.513							
model 3	Ref.	−0.5(−5.1,4.1)	.821	−1.0(−3.6,1.7)	.480							

5:00 to 11:00 h												
model 1	Ref.	0.4(−4.0,4.8)	.858	2.0(−0.6,4.6)	.136							
model 2	Ref.	−0.4(−4.9,4.1)	.866	1.9(−0.8,4.5)	.164							
model 3	Ref.	−0.5(−5.1,4.0)	.812	1.8(−0.8,4.4)	.183							

11:00 to 17:00 h												
model 1	Ref.	0.0(−4.9,4.9)	.998	0.6(−2.3,3.6)	.664							
model 2	Ref.	0.2(−4.9,5.3)	.939	0.8(−2.2,3.8)	.600							
model 3	Ref.	−0.1(−5.2,5.1)	.984	0.7(−2.3,3.7)	.659							

17:00 to 24:00 h												
model 1	Ref.	−0.1(−4.7,4.5)	.974	−2.4(−5.2,0.3)	.081							
model 2	Ref.	0.4(−4.3,5.2)	.857	−2.0(−4.8,0.8)	.155							
model 3	Ref.	0.2(−4.5,4.9)	.930	−2.1(−4.9,0.6)	.132							

β, partial regression coefficient; CI, confidence Intervals;

Model 1 was adjusted for age and community. Model 2 was adjusted further for smoking habit, BMI, family history of diabetes mellitus, exercise habits, skipping breakfast. Model 3 was adjusted further for HbA1c.

* P < 0.05, † P < 0.01, ‡ P < 0.001

## Discussion

This is the first study to show an association between habitual daily alcohol consumption and time-specific glucose levels in daily life. Among males, the time-specific average glucose levels between 5:00 and 11:00 h and between 11:00 and 17:00 h were higher in moderate and heavy drinkers than in never drinkers. The time-specific average glucose levels between 17:00 and 24:00 h were lower in male moderate and heavy drinkers than in never drinkers and female current drinkers.

Possible mechanisms for the lowering of time-specific average glucose levels between 17:00 and 24:00 h among current drinkers could be the suppression of gluconeogenesis, caused by a decrease in the ratio of NAD to NADH [14] and the suppression of growth hormone secretion caused by the acute effect of alcohol consumption. The first mechanism can be explained as follows. Ethanol is oxidized by alcohol dehydrogenase to acetaldehyde and then metabolized to acetic acid by acetaldehyde dehydrogenase. NAD is consumed when ethanol is metabolized to acetaldehyde and acetic acid and thus reduced to NADH in the redox cycles. In an experimental study of five healthy adult males in fasting state, 48 g of ethanol consumption reduced gluconeogenesis by 45% after 5 h compared to no ethanol ingestion [10]. Japanese people generally consume alcohol only at dinner or night; hence, it is speculated that the acute effects of alcohol intake are those that appear between 17:00 and 24:00 h.

As the second mechanism, the acute suppression of growth hormone secretion by alcohol consumption resulted in increased insulin sensitivity and reduced blood glucose levels [15, 16]. Ingestion of 0.8 g/kg of alcohol reduces plasma growth hormone secretion at night by 70–75% of non-drinking baseline levels among healthy males aged 21–26 years [15]. A randomized double-blinded trial demonstrated that consuming gin alone or both gin and tonic, but not tonic alone, results in a marked reduction in plasma growth hormone levels and hypoglycemia within 5 h of drinking [17]. Thus, the abovementioned mechanisms may explain the lower average glucose levels between 17:00 and 24:00 h in current drinkers than in never drinkers in our study.

Contrastingly, the average glucose levels between 5:00 and 11:00 h and between 11:00 and 17:00 h were higher in male moderate and heavy drinkers than in non-drinkers. This can be explained by different physiological processes during the 17:00 to 24:00 post-drinking interval, i.e., increased glucocorticoid levels and sympathetic excitation. Alcohol activates the hypothalamic-pituitary-adrenal axis, resulting in a dose-dependent increase in adrenocorticotropic hormone and glucocorticoid levels [18, 19]. In addition, acetaldehyde, a metabolite of alcohol, acts on the adrenal medulla and sympathetic ganglia to release catecholamines, resulting in sympathetic nerve excitation [20]. In a previous study of 539 Japanese males aged 35–65 years, heavy drinkers (≥46 g ethanol/day) had higher salivary cortisol concentrations and a higher prevalence of blood pressure surge in the morning and higher sympathetic nervous activity and an increased heart rate during both daytime and sleep, than non-drinkers [21]. Since the amount of alcohol metabolized per hour is approximately 100 mg/kg of body weight [22], these hormonal and sympathetic nervous effects can last for 8 h after heavy drinking. Indeed, the long-lasting effects of heavy drinking may become more apparent after the aforementioned acute effects of heavy drinking are attenuated. The higher average glucose levels between 5:00 and 11:00 h and between 11:00 and 17:00 h observed in male moderate and heavy drinkers were probably due to the alcohol-induced increase in glucocorticoid levels and sympathetic nervous system activity.

Furthermore, suppressed glucose levels between 17:00 and 24:00 h were more pronounced in the individual with HbA1c ≤ 5.5% (≤37 mmol/mol), while increased glucose levels between 5:00 and 11:00 h, between 11:00 and 17:00 h were more pronounced in those with HbA1c of 5.6–5.9% (38–41 mmol/mol). The reason may be due to differences in the basal insulin secretory capacity of the pancreas and the insulin resistance of skeletal muscle and liver.

### Strengths and limitations

Our study has several strengths. First, the large sample size enabled us to conduct stratified analyses based on sex and HbA1c levels. Second, our study population was community-dwelling, which ensured the generalizability of our findings. Third, we monitored the daily variation of glucose levels for many days consecutively, which reduced the impact of inter-day variations in glucose levels on the observed association.

The limitations of this study should also be discussed. First, our investigation was cross-sectional; hence, we could not determine the causality between drinking behavior and glucose levels or whether glucose levels affect drinking behavior. However, because our subjects were non-diabetic persons, it is unlikely that glucose levels could affect drinking behavior. Second, we could not examine the association between moderate and heavy drinking in females because of the limited sample size. Third, we did not have the data on alcohol beverage types and time of alcohol consumption, which warrant future studies. Forth, participants in our study may be more health conscious than non-participants, and thus selection bias may exist. Therefore, our results were likely to be generalizable, but not representative of the general population.

## Conclusions

Alcohol consumption was associated with glucose levels in a time-dependent biphasic pattern. It remained uncertain how a time-dependent biphasic patterns of glucose levels by alcohol consumption lead to the development of diabetes mellitus, which will be examined in the future. Circadian changes in glucose levels among nondiabetic persons would provide useful information for the modification of lifestyle in the prevention of diabetes mellitus.

## Abbreviations

BMI: body mass index
DM: diabetes mellitus
FGM: Flash glucose monitoring system
IDF: International Diabetes Federation

## Declarations

### Availability of data and material

Data cannot be shared for privacy or ethical reasons.
